# From Clinical Findings to Treatment Willingness: Self-Perception of Smile Aesthetics Among Dental Students—A Cross-Sectional Study

**DOI:** 10.3390/dj14100624

**Published:** 2026-10-01

**Authors:** Ana Isabel Nicolas-Silvente, Álvaro Jiménez-Guerra, Marta Carazo-Austro, María Dolores Austro-Martínez

**Affiliations:** 1 Department of Stomatology, Faculty of Medicine, CEIR Campus Mare Nostrum, University of Murcia, 30008 Murcia, Spain; 2 Department of Stomatology, Faculty of Dentistry, University of Seville, 41009 Seville, Spain; ajimenez8@us.es; 3 Independent Researcher, 03002 Elche, Spain; martacarazo@correo.ugr.es; 4 Department of Dentistry, CEU Cardinal Herrera University, 03202 Elche, Spain; maria.austromartinez@uchceu.es

**Keywords:** dental education, smile aesthetics, self-perception, treatment willingness, clinical–perceptual agreement, dental students

## Abstract

**Background:** Objective smile characteristics do not necessarily determine how individuals perceive their own dental appearance or whether perceived concerns translate into treatment willingness. This study investigated the relationship between clinical findings, self-perception, and treatment attitude across ten smile characteristics in final-year dental students. **Methods:** This cross-sectional study included 116 students who underwent standardized clinical and photographic assessment and independently evaluated the same smile characteristics and their willingness to receive treatment. Clinical–perceptual agreement was examined using Cohen’s κ and Gwet’s AC1. Multivariable logistic regression assessed whether cumulative self-perceived concerns were associated with overall smile dissatisfaction. **Results:** Sixty-eight participants (58.6%) reported at least one concern. Clinical–perceptual correspondence was feature-dependent. Agreement was high for position/alignment (93.1%; κ = 0.852; AC1 = 0.871) but considerably lower for central-incisor proportion (64.7%; κ = 0.167; AC1 = 0.446); notably, 41 of 48 participants outside the prespecified 77–83% interval reported no concern about tooth size. Treatment willingness was high for concerns about shade and alignment but low for concerns about tooth size and shape. Each additional self-perceived concern was associated with higher odds of overall smile dissatisfaction (adjusted OR = 6.48; 95% CI, 3.13–13.41; *p* < 0.001). **Conclusions:** Clinical deviation, self-perceived concern, and treatment willingness were not interchangeable constructs. Their relationship was strongly feature-dependent, supporting smile assessment that integrates clinical findings with the individual’s perception and treatment preferences.

## 1. Introduction

Dental and gingival features contribute to smile aesthetics, but their clinical measurement does not necessarily predict how a smile is perceived. Differences between professional and lay assessments have been reported for tooth dimensions, gingival display, dental midline, smile arc, and buccal corridors [1,2]. The magnitude of these differences varies according to the feature examined. A deviation from a commonly used aesthetic reference does not, by itself, establish that an individual will regard the feature as problematic.

Dental students provide a useful population in which to examine this issue. During training, exposure to dental morphology, diagnosis, and aesthetic assessment increases, yet comparisons across academic years have produced inconsistent findings. España et al. found no consistent improvement in the detection of smile alterations across academic years, with differences between years showing no linear pattern [3]. Other studies comparing preclinical and clinical students reported differences for selected smile alterations rather than across all characteristics examined [4,5]. Kovačić et al. reported a similar pattern: clinical students were more critical of some modifications but not of every feature [6]. Ntovas et al., in a sample of 410 students from all five academic years, also found differences associated with educational level without a simple linear progression [7]. Dental training therefore cannot be assumed to produce a uniform increase in aesthetic sensitivity.

A methodological issue is also relevant. Much of the available evidence is based on standardized photographs in which individual smile features are digitally altered [3,4,5,6,7]. Such designs permit control of the magnitude of a modification and are well suited to studies of perceptual thresholds. They provide less information about how students evaluate their own teeth. Detecting an altered crown proportion or an occlusal cant in a photograph is not the same as considering a comparable feature problematic in one’s own smile. The smaller body of work addressing self-perception supports this distinction. Ellakany et al. compared perceived and clinically measured incisor shade and examined attitudes toward tooth colour according to dental education [8]. Armalaite et al., using altered smile photographs, also found considerable variation in dental students’ ratings of individual smile characteristics [9].

Studies based on the students’ own dental or smile characteristics are less common. Nguyen et al. used standardized photographs of 216 dental students and related the recorded characteristics to each participant’s satisfaction with his or her own smile. Smile arc, upper-lip curvature, number of displayed teeth, and dental midline were among the characteristics associated with satisfaction [10]. Sharmila et al. examined anterior tooth proportions and found no association between the golden proportion, golden percentage, or RED proportion and self-perceived dental aesthetics [11]. Their sample was small, and the mathematical relationships examined differed from the width-to-height criterion used in this study. Their findings cannot therefore be taken as evidence for or against a particular proportional threshold, but they remain relevant to the distinction between a measurable dental relationship and an individual’s assessment of appearance.

Treatment preference represents a separate question. Dissatisfaction with tooth colour, alignment, or another feature does not necessarily imply a desire for intervention. El Mourad et al. assessed self-perception together with desired treatment in 450 dental students and found bleaching and orthodontic treatment among the options most frequently selected [12]. Similar questions have been examined in other populations. Tin-Oo et al. recorded both satisfaction with dental appearance and desired treatment [13], while Al-Zarea reported associations between dissatisfaction with aspects of dental appearance and interest in aesthetic treatment [14]. These outcomes are related, but dissatisfaction and treatment preference describe different decisions.

Recent data in dental students reinforce this distinction. Nazir et al. found that 72% of participants expressed a desire for some form of aesthetic dental treatment; whitening was selected by 49%, orthodontic treatment by 38.7%, and ceramic veneers by 18.1% [15]. Treatment preference was also associated with psychosocial factors, including dental self-confidence, social impact, and aesthetic concern [15]. That study did not determine whether willingness to undergo treatment was related to an objectively recorded clinical deviation. More generally, the available studies have seldom matched clinical characteristics, self-perception, and treatment preference for the same feature within the same individual.

We examined ten smile characteristics in final-year dental students. For each characteristic, the clinical assessment was matched with the participant’s perception of the same feature and, when concern was reported, with the corresponding treatment attitude. The primary objectives were to assess agreement between clinical findings and self-perception and to examine the relationship between self-perceived concern and willingness to undergo treatment for that characteristic. We also evaluated whether the cumulative number of self-perceived concerns was associated with overall smile dissatisfaction.

## 2. Materials and Methods

### 2.1. Study Design and Participants

A cross-sectional observational study was conducted among final-year dental students at the University of Murcia (Murcia, Spain). All eligible students enrolled in the fifth year of the dental degree were invited to participate, and no student declined participation. The study procedures were explained before enrolment, and written informed consent was obtained from all participants.

The study included 116 participants. Eligibility required healthy, unrestored anterior teeth. Students undergoing active orthodontic treatment or presenting prosthetic restorations or previous restorative treatment involving the anterior aesthetic region, were excluded.

The protocol was reviewed by the Research Ethics Committee of the University of Murcia and received a unanimous favorable opinion on 29 June 2021 (ID 2075/2021). The study was conducted in accordance with the Declaration of Helsinki.

Ten features of the smile were examined: tooth shade, maxillary central-incisor size and proportion, tooth shape, tooth position/alignment, dental midline, occlusal-plane orientation, incisal curvature, buccal corridors, smile line, and gingival zenith. For each feature, the clinical record was considered together with the participant’s perception of that same characteristic and, when dissatisfaction was reported, the treatment the participant would be willing to undergo.

### 2.2. Clinical and Photographic Assessment

Four standardized photographs were obtained from each participant using a Nikon D5200 digital camera (Nikon Inc., New York, NY, USA) and a Sigma 105 mm F2.8 EX DG OS HSM Macro lens (Digital Camera World, Bath, UK). The series comprised an extraoral frontal photograph during a posed smile, an intraoral frontal photograph in maximum intercuspation, and frontal photographs of the maxillary and mandibular dentitions. For the extraoral record, participants were positioned with the Frankfurt horizontal plane and interpupillary line parallel to the floor. The camera was placed at eye level, 60 cm from the participant.

Representative examples of the photographic assessment and the reference guides used for selected smile characteristics are shown in Figure 1.

The posed-smile photograph was used primarily to assess dentolabial relationships, including the dental midline, occlusal-plane orientation, incisal curvature, buccal corridors, and smile line. The intraoral records were used to characterize dental morphology, position/alignment, and gingival architecture.

Tooth shade was assessed independently by three trained observers using the VITA Classical A1-D4 shade guide (VITA Zahnfabrik, Bad Säckingen, Germany). When at least two observers selected the same shade, that value was retained. If all three assessments differed, the lightest of the three recorded shades was used, in accordance with the original examination protocol. In this analysis, shade was retained in its recorded clinical categories and was not divided into aesthetically acceptable and unacceptable ranges.

Photographic measurements were performed by a single examiner using Adobe Photoshop 2020 (Adobe Inc., San Jose, CA, USA). Before the study measurements, the examiner completed a preliminary calibration exercise involving 50 randomly selected cases, each measured three consecutive times. During the study, each measurement was repeated three times, and the value occurring most frequently across the three determinations was retained for analysis. Measurements of the maxillary central incisors were calibrated against direct clinical measurements. Crown height was measured with an Insize 1108-150 electronic digital calliper (insize, Suzhou, Jiangsu, China) from the incisal edge to the gingival zenith, and the corresponding distance on the photograph was used to calibrate the image. Width-to-height ratios were obtained separately for teeth 11 and 21. For this reanalysis, 77–83% was prespecified as a narrow operational interval centered on the approximately 80% maxillary central-incisor width-to-height proportion commonly used in aesthetic assessment. In a standardized-smile experiment, Cooper et al. found an 82% ratio to be the most attractive for normal central incisors and also reported substantial variability in the responses [16]. The 77–83% bounds were therefore used as an operational classification for this dataset rather than as a published universal threshold of attractiveness. A participant was classified as having a proportional deviation when the ratio of either central incisor fell outside this interval.

Tooth shape was recorded as square, oval/ovoid, or triangular. These categories were treated as morphological phenotypes; no category was assigned a priori to an aesthetically acceptable or unacceptable group, and no sex-specific criterion was applied.

Tooth position/alignment was recorded as aligned or malpositioned. Malposition included rotations, versions, and other positional discrepancies documented during the clinical examination. Maxillary dental midline discrepancy was recorded in millimeters relative to the facial reference line. A displacement greater than 4 mm was retained as the study-defined operational threshold. None of the participants exceeded this threshold, so the measured displacement was analyzed without forcing an uninformative binary classification.

Occlusal-plane orientation was evaluated in relation to the intercommissural line and classified as parallel or canted. Incisal curvature was assessed from the relationship between the maxillary incisal edges and the lower lip during the posed smile. A consonant/convex curvature following the lower-lip contour was used as the reference configuration; flat and reverse curves were grouped as non-consonant when a binary classification was required.

Buccal corridors were recorded as normal, absent, or increased/wide. The three original morphological categories were retained for the primary analysis because absent and increased corridors describe different smile configurations and were not assumed to have equivalent perceptual significance.

Smile line was recorded from the position of the upper lip relative to the maxillary dentition during the posed smile. The original low, medium, and high categories were retained, and the single medium-high record present in the source dataset was preserved as recorded rather than reassigned retrospectively. Gingival exposure was also recorded in millimeters. In this analysis, exposure greater than 3 mm was considered a secondary operational threshold. Because only one participant exceeded this value, the categorical smile-line assessment and measured gingival exposure were more informative than a binary classification.

Gingival zenith position was assessed on the frontal intraoral photographs in relation to the longitudinal axis of the anterior teeth. In the original dataset, “Yes” denoted a correct zenith position and “No” an incorrect or altered zenith position; these categories were retained for the present analysis.

### 2.3. Self-Perception and Treatment Attitude

Participants completed a structured questionnaire concerning their perception of their own smile. The questionnaire was completed before participants were informed of the results of the clinical and photographic measurements. Age, sex, previous orthodontic treatment, and overall smile satisfaction were recorded. Overall satisfaction had three response categories: satisfied, dissatisfied, or indifferent.

The questionnaire then addressed the same ten features evaluated clinically. For each feature, participants indicated whether they were satisfied with that aspect of their own smile. A negative response was defined analytically as a self-perceived concern.

Clinical findings and self-perceived concerns were kept separate throughout the analysis. A clinical finding described the characteristic observed or measured during the examination, whereas a self-perceived concern represented the participant’s own appraisal of that characteristic. The presence of a clinical deviation was therefore not assumed to imply dissatisfaction.

When dissatisfaction with a feature was reported, the questionnaire provided treatment alternatives applicable to that characteristic, including the option of receiving no treatment. Selection of an intervention was classified as willingness to undergo treatment. A blank treatment field in the source dataset was interpreted as no treatment, consistent with the coding rules established for the present analysis. The specific treatment selected was retained as a separate variable.

The questionnaire structure allowed each smile feature to be examined across three related levels: clinical finding, self-perceived concern, and treatment attitude. Treatment attitude was evaluated in relation to the corresponding self-perceived concern rather than interpreted as a direct consequence of the clinical finding.

### 2.4. Statistical Analysis

The ten smile features were analyzed individually because their measurement scales and clinical interpretation differed. Categorical variables were summarized as counts and percentages. Continuous variables were described using measures of central tendency and dispersion appropriate to their distribution. Estimates are reported with 95% confidence intervals (95% CIs). No imputation was performed; analyses were based on the available observations for each variable. All tests were two-sided, with the significance level set at 0.05.

Analyses were performed using Stata 19 (StataCorp LLC, College Station, TX, USA). Agreement coefficients, confidence intervals, exact tests, regression models, and sensitivity analyses were implemented using reproducible analytical routines within the same environment.

#### 2.4.1. Clinical Findings and Self-Perception

For features that could be classified clinically into two meaningful categories, the clinical finding and the participant’s perception of that same feature were cross-tabulated at the individual level. Overall agreement was reported together with positive and negative agreement. Cohen’s kappa and its 95% CI were used to quantify agreement beyond chance.

Gwet’s AC1 was calculated as a complementary agreement coefficient because kappa may be difficult to interpret when the marginal distributions are markedly unbalanced. Both coefficients were interpreted together with the underlying cell frequencies and the positive and negative agreement. Confidence intervals for kappa and AC1 were obtained by nonparametric bootstrap resampling at the participant level using 10,000 replicates with a fixed random seed (20260820). Directional discordance between paired classifications was examined with the exact McNemar test where appropriate.

The principal agreement analyses concerned central-incisor size/proportion, position/alignment, occlusal-plane orientation, and incisal curvature. Gingival zenith was examined within the same framework as an exploratory analysis because the less frequent categories contained few observations.

Shade and tooth shape were not assigned artificial binary definitions of clinical normality, and agreement coefficients were therefore not calculated for these variables. Associations between multi-category clinical variables and self-perceived concern were evaluated with the Fisher–Freeman–Halton exact test when contingency tables extended beyond 2 × 2. This approach was applied for tooth shade, tooth shape, buccal corridors, smile-line category, and recorded midline-displacement categories.

The three-category classification was retained for buccal corridors in the primary analysis, with any increased versus non-increased comparison considered secondary. Midline displacement was analyzed from the recorded measurements because no participant exceeded the 4-mm study threshold. Smile-line category and gingival exposure were likewise retained in their recorded forms; analyses using the greater-than-3-mm gingival-exposure threshold were considered exploratory.

#### 2.4.2. Self-Perceived Concern and Treatment Attitude

Treatment attitude was examined separately for each smile feature among participants who reported a concern for that feature. For each characteristic, the number of concerns, the number of participants willing to undergo treatment, and the resulting treatment proportion were calculated. Treatment proportions are reported with 95% Wilson binomial CIs, and the specific treatment options selected were summarized descriptively.

The denominator for each treatment-attitude analysis therefore comprised participants reporting the corresponding concern rather than the complete study sample. Using the feature-specific denominator preserved the conditional structure of the questionnaire and avoided treating multiple treatment decisions contributed by the same participant as independent observations.

#### 2.4.3. Cumulative Self-Perceived Concern and Overall Smile Dissatisfaction

A participant-level concern count was calculated as the number of the ten smile features rated negatively, giving a possible range from 0 to 10. Its association with overall smile dissatisfaction was examined using multivariable binary logistic regression.

Participants who described their overall smile satisfaction as indifferent were excluded from the binary regression analysis rather than being reassigned to either the satisfied or dissatisfied group. Overall smile dissatisfaction was the dependent variable. The number of self-perceived concerns was the principal explanatory variable, with sex and previous orthodontic treatment included as prespecified covariates. Variables were not selected on the basis of univariable *p* values, and no automated or stepwise selection procedure was used.

Associations were estimated using standard maximum-likelihood logistic regression and are reported as odds ratios (ORs) with 95% CIs. Because the number of participants reporting overall dissatisfaction was limited, Firth’s bias-reduced logistic regression was fitted as a sensitivity analysis to assess the stability of the estimates under penalized likelihood.

For families of parallel feature-specific hypothesis tests addressing the same analytical question, the Holm procedure was used as a sensitivity analysis for multiple comparisons. Statistical interpretation emphasized the magnitude and precision of effect and agreement estimates together with the observed data structure, rather than statistical significance alone.

## 3. Results

### 3.1. Study Sample and Overall Smile Satisfaction

The sample comprised 116 final-year dental students (60 women and 56 men), with a mean age of 24.5 ± 3.6 years. Median age was 23 years (interquartile range, IQR, 23–25), and 65 participants (56.0%) had previously undergone orthodontic treatment. Most students were satisfied with their smile overall: 83 (71.55%) answered positively, 27 (23.28%) were dissatisfied, and 6 (5.17%) chose the indifferent response (Table 1).

Sixty-eight students (58.6%) identified at least one of the ten features as a concern. The other 48 reported no concerns. Altogether, there were 128 feature-specific concerns, with individual counts ranging from 0 to 5 (median, 1; IQR, 0–2).

Position/alignment stood out as the feature most often rated negatively, by 42 students (36.2%). Tooth shade was a concern for 20 (17.2%) and tooth shape for 15 (12.9%). The frequencies were lower for dental midline (11, 9.5%), buccal corridors (10, 8.6%), occlusal-plane orientation (8, 6.9%), central-incisor size/proportion (7, 6.0%), incisal curvature (6, 5.2%), smile line (5, 4.3%), and gingival zenith (4, 3.4%).

### 3.2. Clinical Findings and Self-Perception

The extent to which the clinical assessment matched the students’ own perception depended considerably on the feature being examined (Table 2). This was most apparent for central-incisor proportion and position/alignment.

A proportional deviation, defined by a width-to-height ratio outside 77–83% in at least one maxillary central incisor, was found in 48 students (41.4%). Only 7 students, however, regarded tooth size as a concern. Forty-one of the 48 students with a clinical deviation were satisfied with the size of their teeth, while none of the 68 students whose two incisors were within the reference interval reported dissatisfaction.

Agreement was 64.7% for this feature. Cohen’s κ was 0.167 (bootstrap 95% CI, 0.062–0.293) and Gwet’s AC1 was 0.446 (bootstrap 95% CI, 0.263–0.608). Positive agreement was low (25.5%) compared with negative agreement (76.8%). Most disagreement therefore came from students who fell outside the clinical reference interval but did not perceive tooth size as a problem. This asymmetry was confirmed by the exact McNemar test (*p* < 0.001; Holm-adjusted *p* < 0.001).

The findings for position/alignment were different. Clinical malposition was recorded in 44 students. Thirty-nine of them were also dissatisfied with their tooth position, leaving only five with malposition who did not report concern. Of the 72 students classified as aligned, 69 were satisfied and three were not.

Here, agreement reached 93.1%, and the two chance-corrected coefficients were also high: κ = 0.852 (bootstrap 95% CI, 0.744–0.944) and AC1 = 0.871 (bootstrap 95% CI, 0.780–0.951). Positive agreement was 90.7% and negative agreement 94.5%. There was no evidence that the few discordant cases occurred preferentially in either direction (exact McNemar *p* = 0.727).

Seventeen students had a canted occlusal plane. Seven perceived this as a concern, while ten did not. There was also one student with a parallel plane who reported concern. Although overall agreement was 90.5%, κ and AC1 differed appreciably: κ was 0.514 (bootstrap 95% CI, 0.243–0.741), compared with an AC1 of 0.883 (bootstrap 95% CI, 0.803–0.948). A canted plane without a corresponding concern accounted for most of the discordant cases (exact McNemar *p* = 0.0117; Holm-adjusted *p* = 0.023).

The distribution for incisal curvature was comparable in some respects. A non-consonant curve was recorded in 17 students; 6 were concerned about it and 11 were not. None of the 99 students with a consonant/convex curve reported concern about this feature. Overall agreement was 90.5%. Cohen’s κ was 0.482 (bootstrap 95% CI, 0.198–0.715), whereas AC1 was 0.885 (bootstrap 95% CI, 0.803–0.948). The imbalance among discordant pairs remained significant after Holm correction (exact McNemar *p* = 0.001; adjusted *p* = 0.004).

The gingival-zenith analysis involved fewer altered observations and was therefore considered exploratory. Twelve students had an altered zenith position. Four of them reported concern and eight did not; among the 104 students with a correct zenith position, none reported concern. Agreement was 93.1%, with κ = 0.473 (bootstrap 95% CI, 0.129–0.756) and AC1 = 0.921 (bootstrap 95% CI, 0.859–0.971) (Table 3).

Figure 2 brings together the agreement estimates for these five features. The separation between κ and AC1 was greatest for variables with strongly unbalanced marginal distributions. For position/alignment, where the clinical and perceptual classifications corresponded closely, the two coefficients gave similar estimates.

Tooth shade and tooth shape were examined differently because neither had been reduced to a binary definition of clinical normality. Concern about shade was not evenly distributed across the VITA categories (exact Freeman–Halton *p* < 0.001) and occurred mainly among students with A3, A3.5, and B3 shades. Tooth shape was also related to self-perception (*p* < 0.001). None of the students with square teeth reported concern about shape; dissatisfaction was reported by 9 of 53 students with an oval/ovoid form and 6 of 21 with a triangular form.

Differences were also found for buccal corridors (*p* < 0.001). Nine of the 27 students with absent corridors reported concern, as did 1 of the 6 with increased corridors. No concern was recorded among the 83 students with normal corridors.

For smile line, concern was concentrated in the high category (*p* < 0.001): 4 of the 10 students with a high smile line were dissatisfied, compared with 1 of 55 with a low smile line. There were no concerns among students with a medium smile line or in the single medium-high record. The associations observed for shade, shape, buccal corridors, and smile line remained significant after Holm adjustment.

The dental midline produced an unusual distribution. Recorded displacement ranged from 0 to 3 mm, so no student exceeded the prespecified 4-mm threshold. Nonetheless, 11 students considered their midline a concern. Ten had no measurable displacement and the remaining student had a 1-mm deviation. There was no association between concern and the recorded displacement categories (exact Freeman–Halton *p* = 1.000). Gingival exposure >3 mm occurred in only one student, which did not provide enough variation for a meaningful primary binary agreement analysis.

### 3.3. Self-Perceived Concern and Treatment Attitude

Treatment choices provided an additional distinction between the ten features. Reporting dissatisfaction did not necessarily mean that a student wanted the feature treated (Table 4; Figure 3).

For some concerns, willingness to undergo treatment was very high. All 8 students concerned about occlusal-plane orientation selected a treatment option. The corresponding proportions were 39/42 for position/alignment (92.9%; 95% CI, 81.0–97.5), 10/11 for dental midline (90.9%; 95% CI, 62.3–98.4), and 18/20 for tooth shade (90.0%; 95% CI, 69.9–97.2).

This was not the case for tooth dimensions or morphology. Among the 7 students concerned about central-incisor size/proportion, only one selected treatment (14.3%; 95% CI, 2.6–51.3). For tooth shape, treatment was selected by 2 of 15 students (13.3%; 95% CI, 3.7–37.9). All four students who reported concern about gingival zenith chose no treatment.

The remaining features occupied an intermediate position. Four of six students concerned about incisal curvature were willing to undergo treatment (66.7%), as were 6 of 10 concerned about buccal corridors (60.0%) and 3 of 5 concerned about smile line (60.0%).

These differences are shown in Figure 3. The first panel gives the frequency with which each feature was perceived as a concern in the full sample; the second shows treatment willingness only among students who reported that concern. Position/alignment was both frequently perceived negatively and frequently associated with treatment selection. Tooth size and shape showed a different pattern: when concern was present, it seldom led to a treatment choice.

### 3.4. Number of Concerns and Overall Smile Dissatisfaction

Six students who selected the indifferent response for overall smile satisfaction were excluded from the binary regression analysis. The model therefore included 110 participants, of whom 83 were satisfied and 27 dissatisfied.

The number of individual concerns was strongly related to overall dissatisfaction. After adjustment for sex and previous orthodontic treatment, the odds of being dissatisfied with the smile increased by a factor of 6.48 for each additional feature rated negatively (OR, 6.48; 95% CI, 3.13–13.41; *p* < 0.001).

There was no evidence of an independent association with either of the other two variables in the model. The OR for female sex was 0.85 (95% CI, 0.24–3.02; *p* = 0.797), and that for previous orthodontic treatment was 0.59 (95% CI, 0.17–2.13; *p* = 0.424).

Because the number of dissatisfied participants was limited, the model was repeated using Firth’s bias-reduced logistic regression. The estimate for the number of concerns remained similar (OR, 5.54; 95% CI, 2.85–10.76; *p* < 0.001). The corresponding estimates for female sex (OR, 0.85; 95% CI, 0.25–2.88) and previous orthodontic treatment (OR, 0.61; 95% CI, 0.18–2.06) were likewise close to those obtained with maximum-likelihood estimation.

Figure 4 shows the adjusted probability of overall smile dissatisfaction according to the number of self-perceived concerns.

## 4. Discussion

### 4.1. Principal Findings

Clinical assessment and personal appraisal were closely aligned for some smile features but not for others. The clearest contrast was between position/alignment and central-incisor proportion. Treatment preference also varied by feature: willingness to intervene was high for several concerns but low for tooth size and shape. At the participant level, overall smile dissatisfaction increased as the number of negatively perceived features increased.

Previous studies in dental students also suggest that smile characteristics are not perceived with equal intensity. El Mourad et al. examined self-perceived dental aesthetics together with desired treatment and found bleaching and orthodontic treatment among the interventions most frequently desired [12]. Armalaite et al. reported strong effects of hypodontia, gingival smile, reversed occlusal-plane curvature, and dental crowding on attractiveness ratings [9]. Studies comparing educational levels have likewise found feature-specific differences rather than a uniform change across all smile characteristics [3,4,5,6,7,17,18]. These observational comparisons, however, do not isolate the effect of training.

Most previous studies have used standardized or digitally modified smiles to investigate aesthetic perception and compare observer groups or educational levels [1,3,4,6,19,20]. The present study addressed a different question: participants evaluated features of their own smiles, and these judgements were paired with the corresponding clinical record. Nguyen et al. related smile satisfaction to clinical smile characteristics in dental students [10]. The present analysis extends that approach by examining agreement and discordance within individuals and by showing that the relationship between clinical status and personal concern varies by feature.

### 4.2. Clinical Findings and Self-Perception

The largest mismatch involved maxillary central-incisor proportion. Forty-eight students had at least one central incisor outside the prespecified 77–83% width-to-height interval, yet only seven considered tooth size a concern. Thus, 41 students had a proportional deviation without a corresponding negative perception. The low positive agreement and low kappa reflect this distribution. In this sample, a morphometric deviation was not equivalent to an aesthetic complaint.

This interpretation is consistent with experimental perception research. Kokich et al. showed that orthodontists, general dentists, and laypeople did not respond identically to altered dental aesthetics [19], and their subsequent study of symmetric and asymmetric changes again demonstrated differences between professional and lay judgements [21]. Ker et al. reported relatively broad ranges of acceptability for several smile features from the lay perspective [20], while McLeod et al. documented population-related differences in acceptable smile characteristics [22]. These studies provide context for the present finding but do not establish the 77–83% interval as a perceptual threshold. A numerical departure from an aesthetic reference therefore needs to be considered alongside the individual’s perception.

Alignment showed a different pattern. Clinical malposition was present in 44 students, 39 of whom reported concern about tooth position; only three students classified as aligned reported concern. Agreement was high by both kappa and AC1. Alignment has also been identified repeatedly in studies of dental appearance. Armalaite et al. reported unfavourable ratings for crowding [9], while Samorodnitzky-Naveh et al. identified poor alignment among the main reasons for dissatisfaction in young adults [23]. Studies combining satisfaction with desired aesthetic treatment have similarly implicated alignment [13,14]. Here, the association was observed within the same individuals, rather than inferred from separate group-level frequencies.

Occlusal-plane orientation and incisal curvature occupied an intermediate position. Overall agreement exceeded 90% for both, but several students with a clinical deviation did not report concern. The difference between kappa and AC1 is relevant because the marginal distributions were markedly unbalanced. Experimental work has shown that occlusal cant, smile arc, and related changes can influence attractiveness ratings, although sensitivity varies with the magnitude of the alteration and the observer [1,3,20,22,24,25,26,27,28]. Parekh et al. found effects of smile arc and buccal-corridor space in ratings by orthodontists and laypersons [22], and Al Taki et al. reported differences among orthodontists, dentists, and laypersons when altered smile characteristics were assessed [1]. These studies concern judgements of other people’s smiles and therefore provide context rather than a direct comparison with self-perception.

Cohen’s kappa can be affected by highly unbalanced prevalence or marginal distributions. Wongpakaran et al. found Gwet’s AC1 to be less affected by prevalence and marginal probabilities in a direct comparison with kappa [29], while Tan et al. described similar behaviour of kappa in the presence of a highly prevalent feature [30]. Neither coefficient was therefore considered in isolation. The contingency table, observed agreement, positive and negative agreement, kappa, and AC1 were considered together, particularly for occlusal-plane orientation, incisal curvature, and gingival zenith.

The dental midline presented a different pattern. No participant exceeded the study threshold of 4 mm, yet 11 students reported concern; ten had no measurable horizontal displacement and one had a 1-mm deviation. Experimental perception studies have reported relatively high tolerance for small midline shifts. Cracel-Nogueira and Pinho found maxillary midline shift to be the least perceptible of the modifications they tested [31], while Pinho et al. showed that dental asymmetries were judged differently by laypersons, orthodontists, and prosthodontists [32]. Parrini et al. likewise identified feature-specific perceptual thresholds in their systematic review [33]. In this sample, the questionnaire response labelled “midline” did not correspond closely to measured horizontal displacement and may instead have reflected a broader perception of dental or smile asymmetry. Because other components of asymmetry were not assessed, this interpretation cannot be verified from the present data.

Buccal corridors were also difficult to reduce to a single binary aesthetic classification. None of the 83 students with normal corridors reported concern, whereas concern occurred with both absent and increased corridors. Experimental studies show that corridor width can influence smile ratings [34], but preferred or tolerated configurations vary across studies and observer groups [1,2,20,22,24,35,36]. Retaining the three recorded categories preserved information that would have been lost by combining absent and increased corridors into a single abnormal group.

For smile line, concern was concentrated in the high category: four of ten students with a high smile line reported concern, compared with one of 55 with a low smile line and none with a medium smile line. Manipulation studies have repeatedly shown that gingival display influences smile ratings, although the point at which it becomes objectionable varies among studies and evaluators [1,2,20,22,33,35,36]. Only one participant had gingival exposure greater than 3 mm, so the threshold is used here only as an operational descriptor.

### 4.3. From Self-Perceived Concern to Treatment Willingness

Treatment responses add information that is not captured by dissatisfaction alone. The contrast was strongest for tooth size and shape: only one of seven students concerned about size and two of 15 concerned about shape selected treatment. By comparison, 90% of those concerned about shade and 92.9% of those concerned about alignment chose an intervention. Concern about the dental midline and occlusal-plane orientation was also usually followed by treatment selection.

Colour and alignment have been described both as sources of dissatisfaction and as targets of desired aesthetic treatment. Samorodnitzky-Naveh et al. reported tooth colour as the leading reason for dissatisfaction in their young-adult sample, with alignment also contributing [23]. Tin-Oo et al. assessed satisfaction with dental appearance alongside desired treatments [13], while Al-Zarea reported associations between dissatisfaction with aspects of dental appearance and desired aesthetic treatment [14]. Akarslan et al. likewise examined received and desired treatment in relation to dental aesthetic satisfaction [37]. The populations and questionnaires are not directly comparable with those used here, but the recurrence of shade and alignment is consistent with the high treatment willingness observed for both features.

Bleaching and orthodontic treatment are also prominent in studies of dental students. El Mourad et al. found both among the most desired interventions [12]. Nazir et al. reported whitening as the most frequently desired aesthetic treatment, followed by orthodontic treatment and ceramic veneers, and also examined the psychosocial context of those preferences [15]. In the present questionnaire, the treatment response was linked directly to a specific concern in the student’s own smile. This allowed treatment choice to be examined in relation to that concern.

A concern did not always lead to treatment selection. The reasons cannot be reconstructed from the dataset. Expected benefit, biological cost, treatment duration, financial cost, perceived severity, or familiarity with a procedure may influence the decision, but none of these factors was measured. The results show that willingness to undergo treatment differed markedly among smile features, even when a concern was reported.

Experimental research also supports separating aesthetic appraisal from intervention preference. Sabri et al. assessed attractiveness and perceived treatment need for maxillary midline diastemas of different widths in laypersons, dental students, and dentists [38]. Their design differs from the present study, but the findings are relevant because they also distinguish aesthetic appraisal from the decision to seek treatment.

### 4.4. Cumulative Concerns and Overall Smile Dissatisfaction

At the participant level, the cumulative number of concerns was strongly associated with overall smile dissatisfaction. Each additional negatively perceived feature was associated with substantially higher odds of dissatisfaction after adjustment for sex and previous orthodontic treatment. The Firth estimate was similar in magnitude, although the confidence intervals remained wide.

This association is compatible with the broader psychosocial literature on dental appearance. Klages et al. developed the PIDAQ to assess dental self-confidence, social impact, psychological impact, and aesthetic concern [39]. Subsequent studies in university and student populations have linked dental aesthetics with psychosocial well-being, dental self-confidence, perfectionism, or self-esteem [40,41,42,43]. AlSagob et al. likewise examined self-perceived dental aesthetics in relation to psychosocial well-being and dental self-confidence among university students [44]. These studies place the present finding within the broader literature on subjective dental appearance, but they do not validate the concern count as a psychological scale.

The concern count records the number of the ten smile features rated negatively. It is not a psychometric instrument and should not be used as a substitute for PIDAQ or another validated measure. Participants reporting more negatively perceived features were more likely to be dissatisfied with their smile overall. Because the study was cross-sectional, the direction of this relationship cannot be determined.

Neither sex nor previous orthodontic treatment was independently associated with overall dissatisfaction after the concern count was included in the model. Previous studies have reported mixed findings regarding sex or gender and aesthetic perception [3,6,9,35]. Cracel-Nogueira and Pinho also found no difference in perception between laypersons with and without previous orthodontic treatment in their sample [31]. The finding here is specific to this model and cohort.

### 4.5. Educational and Clinical Implications, Strengths, and Limitations

All participants were final-year dental students, so the sample represents an advanced dental-student population. The cohort is relevant to questions of professional perception, but it does not allow the effect of dental education itself to be estimated. There was no non-dental control group and no longitudinal follow-up across academic years. Observational studies across educational levels have reported differences in the perception of selected smile features [3,4,5,6,7], but they do not isolate a causal effect of training. The findings describe self-perception in this population rather than the effect of dental education.

For education, a clinical reference value and a patient’s treatment goal should be considered separately. This was particularly evident for central-incisor proportion, where many students fell outside the selected interval without considering tooth size problematic. Alignment showed almost the reverse pattern, with close clinical–perceptual agreement and frequent treatment willingness. In teaching aesthetic assessment, measurement of a feature can therefore be kept distinct from whether the patient considers it problematic and from whether intervention is desired. This distinction can be incorporated through structured case exercises in which students first record the clinical finding without assigning treatment need, then elicit the patient’s perception of the same feature, and finally discuss treatment options in relation to the patient’s goals. Comparing cases with similar measurements but different patient concerns may help students recognize that aesthetics reference values inform diagnosis but do not determine treatment demand. Assessment rubrics could score clinical measurement, patient-centred communication, and justification of treatment separately.

The study has several strengths. Clinical findings, self-perception, and treatment attitude were linked within the same individual for the same ten characteristics. This permitted the direction of disagreement to be examined rather than relying on separate aggregate percentages. Variables were retained in their original morphological categories when a defensible binary aesthetic threshold was lacking. For the binary analyses, agreement was summarized using complementary measures rather than kappa alone.

Several limitations should be considered. The cross-sectional design limits causal interpretation. Although all eligible students in the cohort were invited and none declined participation, the sample was drawn from a single institution and was not probability-based. Thus, mom-response bias within the invited cohort is unlikely, but selection related to the institutional, educational, and cultural setting may limit generalizability. Cultural and educational context may influence aesthetic judgements, and previous work has shown variation in acceptable smile characteristics across populations and observer groups [22,32,37,38]. Formal intraexaminer repeatability coefficients for the photographic measurements and interobserver reliability statistics for shade assessment were not available for retrospective analysis. Repeating each photographic measurement three times reduced reliance on a single determination but does not replace a formal measurement-error or reliability study.

Treatment preference was measured in simple terms. Participants indicated whether they would accept treatment for a feature, but the study did not measure the strength of that preference or the reasons for the choice. The treatment menus were feature-specific, so differences in treatment willingness between features should be interpreted descriptively rather than as direct estimates of the propensity of a concern to generate treatment demand. The operational criteria used here should not be treated as universal thresholds separating an aesthetic from a non-aesthetic smile. The available perception literature documents substantial variation in acceptable thresholds across smile features, populations, and evaluator groups [13,14,15,19,20,21,22,31,32,37,38,39].

The clinical meaning of a finding depends on the feature and on how the individual views his or her own smile. Clinical measurement remains essential, but these results suggest that it should be considered together with the individual’s assessment and treatment preference rather than treated as equivalent to perceived treatment need.

## 5. Conclusions

Among final-year dental students, clinical findings did not consistently correspond to personal aesthetic concern across the smile features examined. Clinical–perceptual correspondence was feature-specific, ranging from close agreement for alignment to marked discordance for maxillary central-incisor proportion.

Treatment preference provided an additional level of interpretation: some concerns were commonly followed by willingness to intervene, whereas others were not. Overall dissatisfaction was also associated with the accumulation of self-perceived concerns. Aesthetic assessment should therefore distinguish between the feature identified clinically, the feature the individual regards as problematic, and the change that the individual is willing to pursue.

## Figures and Tables

**Figure 1:**
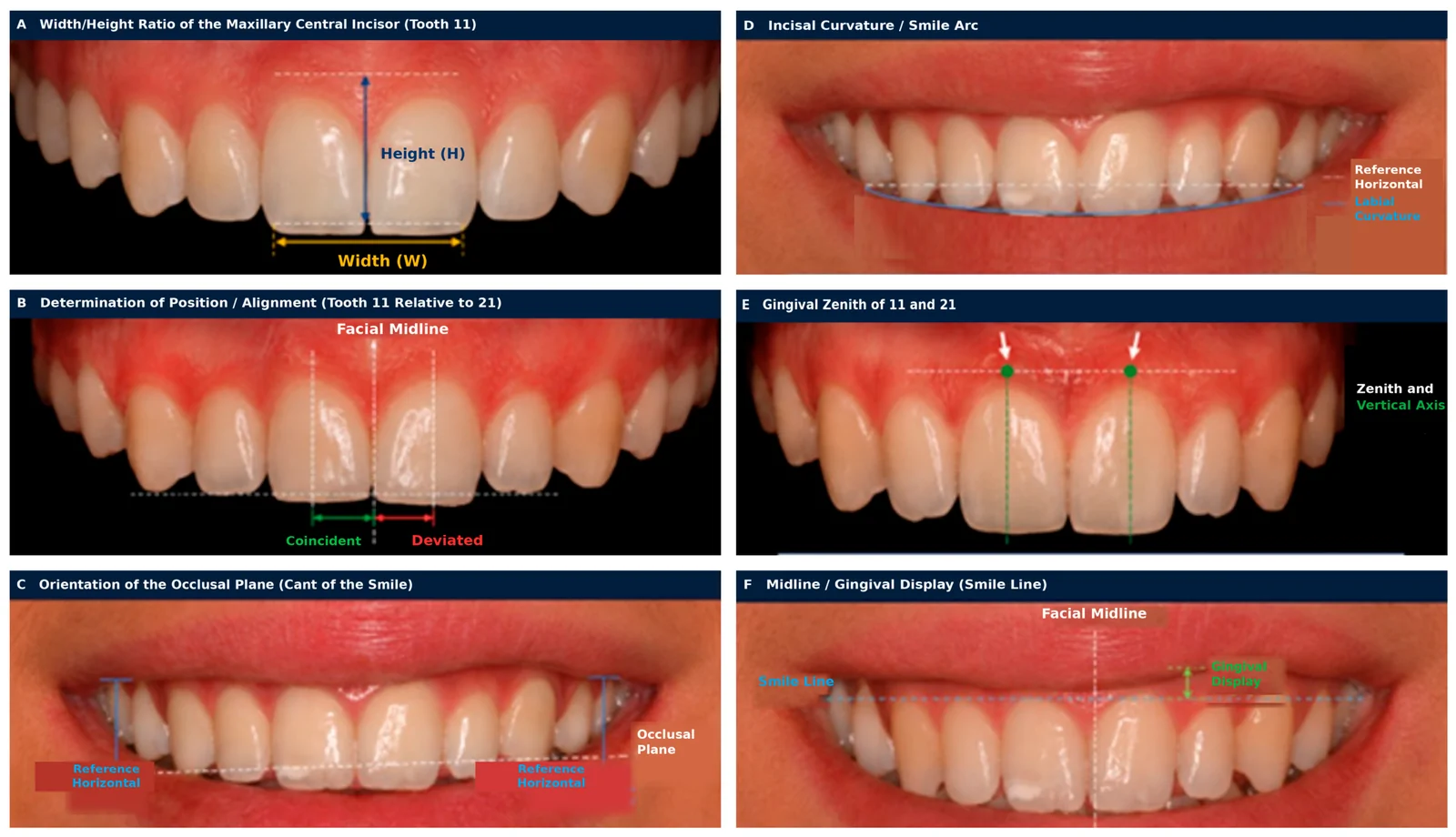
Representative photographic assessment of selected smile characteristics. (**A**) Maxillary central-incisor width-to-height ratio, illustrating the operational 77–83% reference interval used in this study; arrows indicate the width and height measurements. (**B**) Assessment of tooth position/alignment; vertical reference lines indicate the facial midline and the relative position of the maxillary central incisors. (**C**) Orientation of the occlusal plane relative to the horizontal reference: reference lines illustrate the assessment of occlusal-plane cant. (**D**) Incisal curvature/smile arc; colored curves illustrate the reference patterns used to classify the relationship between the maxillary incisal curvature and the lower-lip contour. (**E**) Gingival zenith assessment; dots indicate the zenith points and reference lines illustrate their vertical comparison. (**F**) Dental/facial midline and gingival display (smile line); reference lines indicate the facial midline and lip level used for assessment of midline deviation and gingival display. Arrows, dots, solid and dotted lines, and colors are used as visual measurement and reference guides, as specified within each panel.

**Figure 2:**
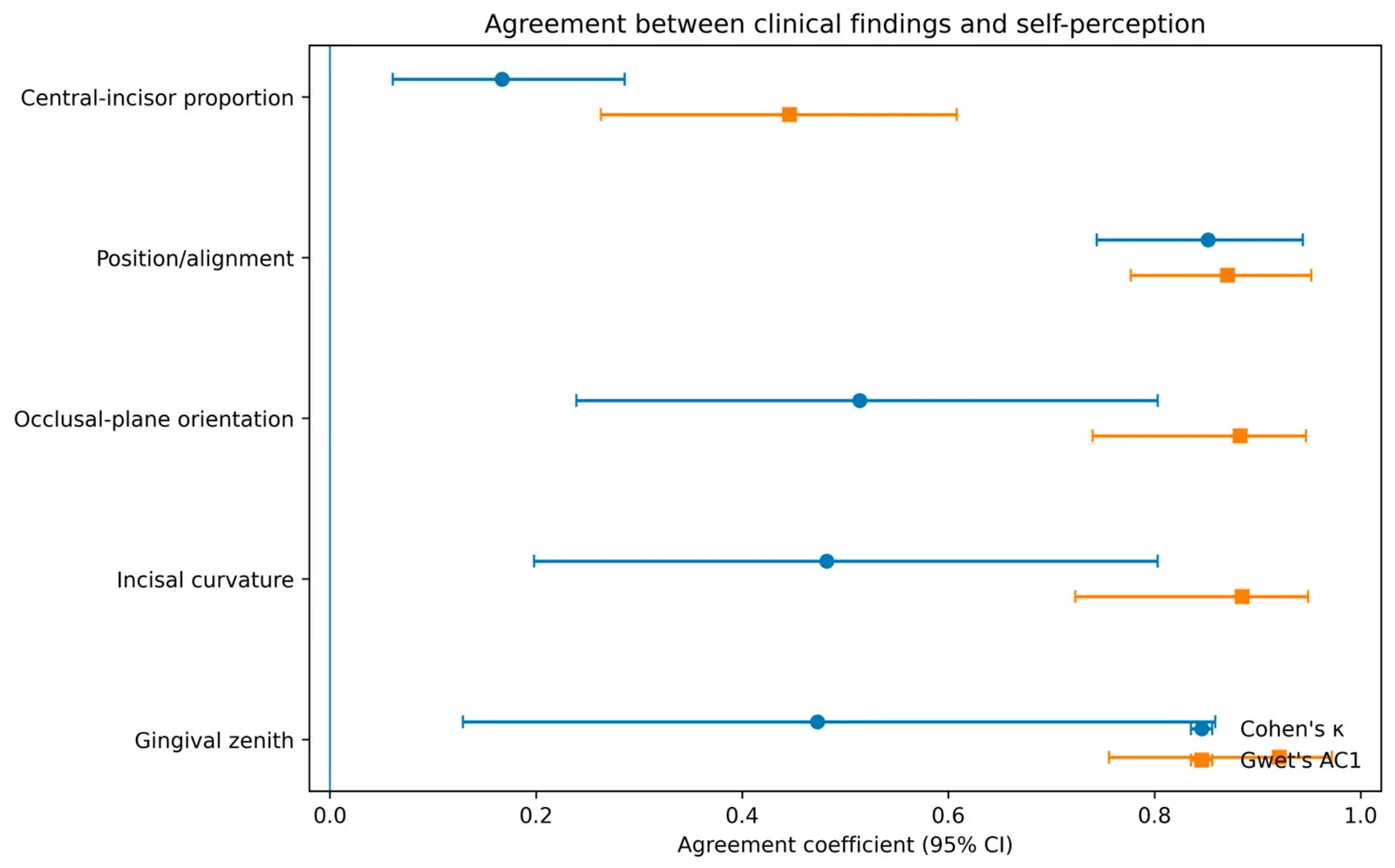
Agreement between clinical findings and self-perception. Cohen’s κ and Gwet’s AC1 with 95% confidence intervals for the five features analyzed using binary clinical and perceptual classifications. Confidence intervals were obtained by participant-level nonparametric bootstrap with 10,000 replicates using a fixed random seed (20260820). The analysis of gingival zenith was exploratory.

**Figure 3:**
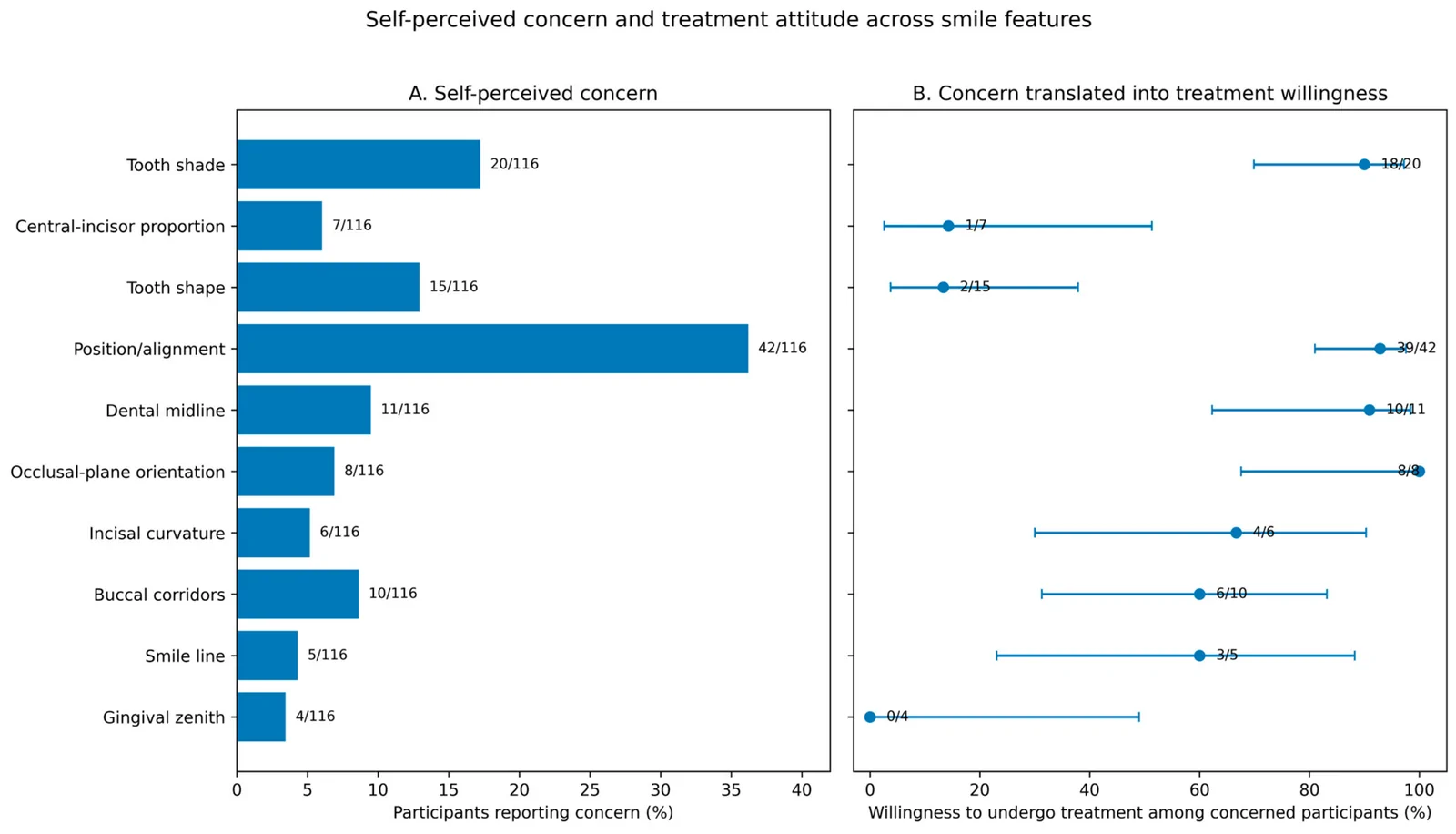
Self-perceived concern and treatment attitude across smile features. (**A**) Percentage of the 116 participants reporting concern for each feature. Labels show the corresponding number of participants. (**B**) Percentage willing to undergo treatment among participants who reported concern for that feature. Error bars indicate 95% Wilson binomial confidence intervals.

**Figure 4:**
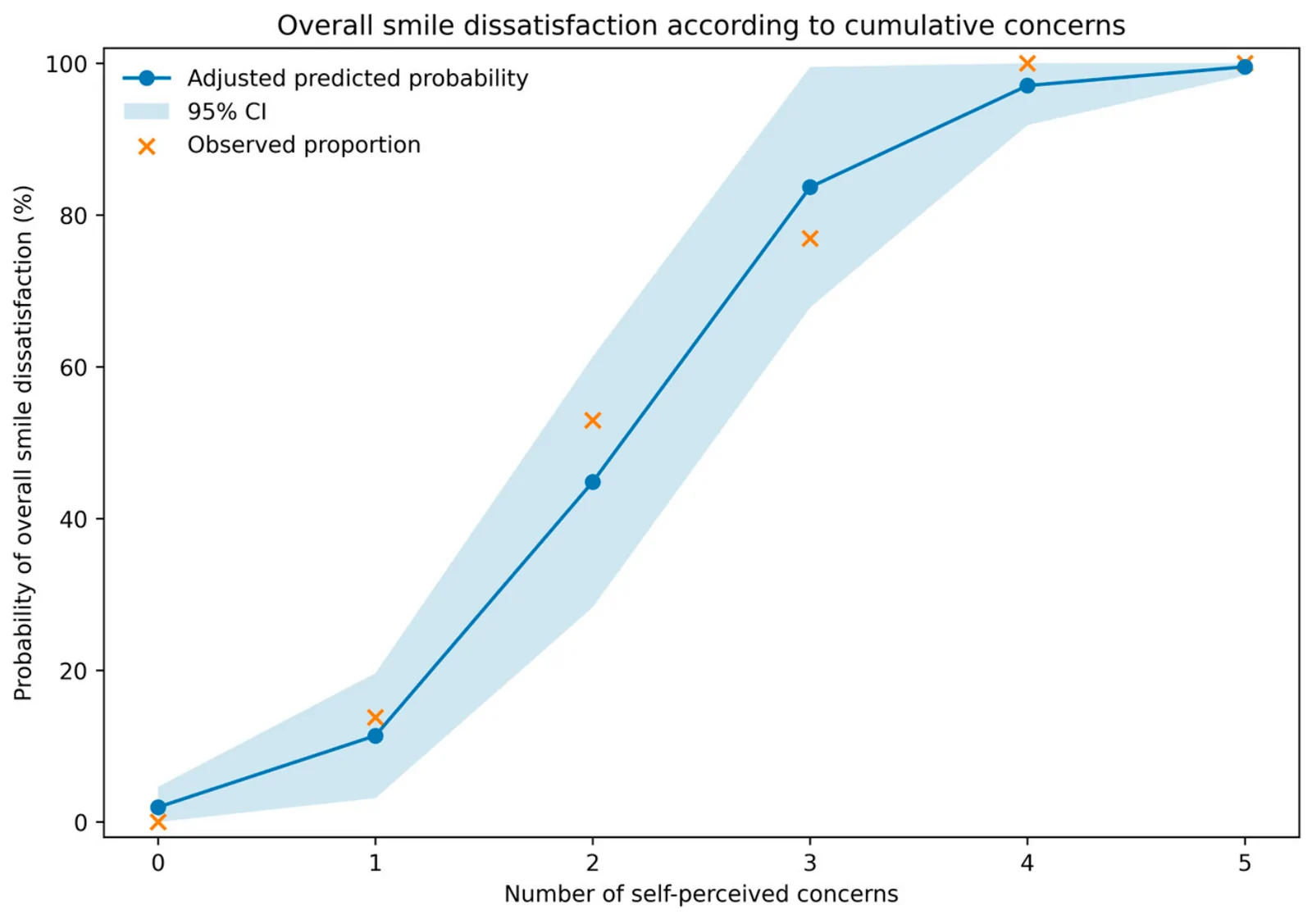
Adjusted probability of overall smile dissatisfaction according to the number of self-perceived concerns. Predicted probabilities were obtained from the multivariable logistic regression model adjusted for sex and previous orthodontic treatment. The shaded band represents the 95% confidence interval; observed proportions are shown for comparison.

**Table 1:** Participant characteristics, overall smile satisfaction, and cumulative self-perceived concerns.

Characteristic	Value
Age, years	24.5 ± 3.6; median 23 (IQR 23–25)
Sex	
Female	60 (51.7)
Male	56 (48.3)
Previous orthodontic treatment	65 (56.0)
Overall smile satisfaction	
Satisfied	83 (71.55)
Dissatisfied	27 (23.28)
Indifferent	6 (5.17)
Participants with ≥1 self-perceived concern	68 (58.6)
Concern count	median 1 (IQR 0–2); range 0–5

**Table 2:** Distribution of clinical findings and corresponding self-perceived concerns across the ten smile characteristics. Concern percentages are calculated within each clinical category.

Feature	Clinical Category	*n* (%)	Concern, *n* (%)
Tooth shade	A1	15 (12.9)	0 (0.0)
	A2	43 (37.1)	3 (7.0)
	A3	19 (16.4)	9 (47.4)
	A3.5	4 (3.4)	3 (75.0)
	B1	11 (9.5)	0 (0.0)
	B2	14 (12.1)	1 (7.1)
	B3	5 (4.3)	4 (80.0)
	C1	3 (2.6)	0 (0.0)
	C2	2 (1.7)	0 (0.0)
Central-incisor proportion	Within 77–83% range bilaterally	68 (58.6)	0 (0.0)
	Outside 77–83% range in ≥1 central incisor	48 (41.4)	7 (14.6)
Tooth shape	Square	42 (36.2)	0 (0.0)
	Ovoid/oval	53 (45.7)	9 (17.0)
	Triangular	21 (18.1)	6 (28.6)
Position/alignment	Aligned	72 (62.1)	3 (4.2)
	Malposition	44 (37.9)	39 (88.6)
Dental midline	0 mm	95 (81.9)	10 (10.5)
	0.5 mm	6 (5.2)	0 (0.0)
	1 mm	13 (11.2)	1 (7.7)
	2 mm	1 (0.9)	0 (0.0)
	3 mm	1 (0.9)	0 (0.0)
Occlusal plane	Canted	17 (14.7)	7 (41.2)
	Parallel	99 (85.3)	1 (1.0)
Incisal curvature	Consonant/convex	99 (85.3)	0 (0.0)
	Non-consonant	17 (14.7)	6 (35.3)
Buccal corridors	Increased	6 (5.2)	1 (16.7)
	Absent	27 (23.3)	9 (33.3)
	Normal	83 (71.6)	0 (0.0)
Smile line	High	10 (8.6)	4 (40.0)
	Low	55 (47.4)	1 (1.8)
	Medium	50 (43.1)	0 (0.0)
	Medium-high	1 (0.9)	0 (0.0)
Gingival zenith	Altered zenith position	12 (10.3)	4 (33.3)
	Correct zenith position	104 (89.7)	0 (0.0)

**Table 3:** Agreement between binary clinical findings and self-perception. Overall, positive, and negative agreement are shown together with Cohen’s κ and Gwet’s AC1 (95% bootstrap CIs), exact McNemar *p* values, and Holm-adjusted *p* values. Gingival zenith was analyzed exploratorily.

Feature	Overall %	Positive %	Negative %	Cohen’s κ (95% CI)	Gwet AC1 (95% CI)	McNemar *p*	Holm *p*
Central-incisor proportion	64.7	25.5	76.8	0.167 (0.062–0.293)	0.446 (0.263–0.608)	<0.001	<0.001
Position/alignment	93.1	90.7	94.5	0.852 (0.744–0.944)	0.871 (0.780–0.951)	0.727	0.727
Occlusal-plane orientation	90.5	56.0	94.7	0.514 (0.243–0.741)	0.883 (0.803–0.948)	0.012	0.023
Incisal curvature	90.5	52.2	94.7	0.482 (0.198–0.715)	0.885 (0.803–0.948)	<0.001	0.004
Gingival zenith	93.1	50.0	96.3	0.473 (0.129–0.756)	0.921 (0.859–0.971)	0.008	0.023

**Table 4:** Treatment willingness among participants reporting a self-perceived concern for each smile characteristic. Percentages use the number reporting the corresponding concern as the denominator; 95% CIs are Wilson binomial confidence intervals.

Feature	Concern *n*	Treatment *n*	Treatment % (95% CI)	Selected Options, *n*
Tooth shade	20	18	90.0 (69.9–97.2)	Bleaching 17, No treatment 2, Veneers 1
Central-incisor size/proportion	7	1	14.3 (2.6–51.3)	No treatment 6, Veneers 1
Tooth shape	15	2	13.3 (3.7–37.9)	No treatment 13, Veneers 2
Position/alignment	42	39	92.9 (81.0–97.5)	Orthodontics 35, Veneers 4, No treatment 3
Dental midline	11	10	90.9 (62.3–98.4)	Orthodontics 10, No treatment 1
Occlusal-plane orientation	8	8	100.0 (67.6–100.0)	Orthodontics 7, Veneers 1
Incisal curvature	6	4	66.7 (30.0–90.3)	Orthodontics 4, No treatment 2
Buccal corridors	10	6	60.0 (31.3–83.2)	Orthodontics 6, No treatment 4
Smile line	5	3	60.0 (23.1–88.2)	No treatment 2, Orthodontics 2, Crown lengthening 1
Gingival zenith	4	0	0.0 (0.0–49.0)	No treatment 4

## Data Availability

The de-identified dataset supporting the findings of this study is publicly available in Zenodo at https://doi.org/10.5281/zenodo.22093577 (accessed on 12 September 2026). The dataset contains the variables required to reproduce the principal analyses reported in this article. Exact participant age and other potentially identifying information were removed or grouped to reduce re-identification risk. Clinical photographs are not included in the public dataset.
